# Cliques within the crowd: identifying medical conference attendee subgroups by their motivations for participation

**DOI:** 10.1007/s10459-023-10220-3

**Published:** 2023-04-29

**Authors:** Sai Sreenidhi Ram, Daniel Stricker, Carine Pannetier, Nathalie Tabin, Richard W. Costello, Daiana Stolz, Kevin W. Eva, Sören Huwendiek

**Affiliations:** 1 Department for Assessment and Evaluation, Institute for Medical Education (IML), University of Bern, Mittelstrasse 43, Bern, Switzerland; 2 Graduate School for Health Sciences (GHS), University of Bern, 3012 Bern, Switzerland; 3 European Respiratory Society, Lausanne, Switzerland; 4 Department of Respiratory Medicine, Royal College of Surgeons, Dublin, Ireland; 5 The Clinics of Respiratory Medicine and Pulmonary Cell Research, University Hospital Basel, Basel, Switzerland; 6 Clinic of Respiratory Medicine, Faculty of Medicine, University of Freiburg, Freiburg, Germany; 7 Centre for Health Education Scholarship, University of British Columbia, Vancouver, Canada

**Keywords:** Attendee subgroups, Conference attendees, Conferences, Motivations, Virtual conference

## Abstract

Conferences enable rapid information sharing and networking that are vital to career development within academic communities. Addressing diverse attendee needs is challenging and getting it wrong wastes resources and dampens enthusiasm for the field. This study explores whether, and how, motivations for attendance can be grouped in relation to preferences to offer guidance to organizers and attendees. A pragmatic constructivist case study approach using mixed methods was adopted. Semi-structured interviews completed with key informants underwent thematic analysis. Survey results outlining attendees’ perspectives underwent cluster and factor analysis. Stakeholder interviews (n = 13) suggested attendees could be grouped by motivations predictable from level of specialisation in a field and past engagement with conferences. From n = 1229 returned questionnaires, motivations were clustered into three factors: learning, personal and social. Three groups of attendees were identified. *Group *1 (n = 500; 40.7%) was motivated by all factors. *Group *2 (n = 345; 28.1%) was mainly motivated by the learning factor. *Group *3 (n = 188; 15.3%) scored the social factor highest for in-person conferences and the learning factor highest for virtual meetings. All three groups expressed a preference for hybrid conferences in the future. This study indicates that medical conference attendees can be clustered based on their learning, personal and social motivations for attendance. The taxonomy enables organizers to tailor conference formats with guidance on how to utilize hybrid conferences, thereby enabling better catering to attendees’ desires for knowledge gain relative to networking.

## Introduction

“In crowds we have unison, in groups harmony. We want the single voice but not the single note; that is the secret of the group.” – Tonn J (1991)

Despite conferences playing a vital role in the health and professional development of academic communities, considerable challenges exist for organizers. Always prominent has been that potential delegates are motivated to attend for a wide variety of reasons, requiring programs that appear to meet broad interests and multiple needs. Getting it wrong risks suboptimal engagement and wasting resources. The emergence of COVID-19 and related pandemic measures only served to amplify such problems. All were forced to cancel meetings or transition to a virtual platform, thereby pushing organizers, presenters, attendees and the larger academic community into uncharted territory (Lessing et al, 2020). As the world re-opens, all stakeholders in academic communities have been forced to grapple with the cost, benefit and risk ratios inherent in conference attendance in ways that were often taken for granted previously. Contemplating these challenges would be more manageable if we had a clearer sense of whether and how subgroups of attendees can be identified in relation to their demographics and needs. Investigating those questions may help organizers better cater to delegates when planning future meetings as well as providing a reflective tool for delegates themselves to determine on which activities to spend their limited funds.

What is known within the medical conference context is that some attendees go to meetings to present work to colleagues and obtain feedback (Goodhand et al., 2011), some go to promote and facilitate collaborative work and growth (European Society of Radiology, 2020), and some go for the sake of advancing their own learning (often to satisfy professional continuing medication education requirements). More extensive research on conference motivations has been conducted within the business and tourism industry (e.g., Mair et al., 2018; Rogers & Davidson, 2015). Motivations reported there include opportunities for face-to-face meetings with colleagues (Layng, 2009), personal and professional development (Yoo & Chon, 2008), networking (Mair & Frew, 2018), collaboration (Hixson, 2012), learning and knowledge sharing (Rogers, 2013) and presenting one’s work (Venkatesh et al., 2000). More personal considerations include conference location (Yoo & Zhao, 2010), time, convenience, health, security and affordability (, 2010). Clustering studies suggest differences in delegate motivations based on age, gender, and education level (Mair, 2010).

With the generation of new conference formats (both virtual and hybrid), questions arise as to whether the motivations and preferences previously expressed simply reflect what seemed possible at the time (i.e., what had already been experienced) and whether they capture how meetings might need to continue to evolve in the future. With a disruption to long-established financial and logistical routines, examining participant motivations for conferences during this new era offers an important step for determining how to best support the full breadth of academics moving forward. Taken together, pre-existing literature, ongoing technical innovation, environmental pressures and financial constraints sum to indicate we need a better understanding of what motivates delegates to attend conferences, whether clusters of motivation exist and whether those clusters relate to sociodemographic or experience levels. Such information would be invaluable to enable organizers, academic leaders and potential delegates themselves to optimize their efforts towards ongoing advancements in the academic communities. Therefore, our study is aimed at addressing two main research questions:

1. Do attendees’ motivations for conference attendance cluster together in ways that suggest subgroups of participants?
2. Are any such subgroups associated with attendee demographics, experiences with past in-person conference attendance, satisfaction with virtual conference formats, or preference for future conferences?

## Methods

### Overview of study design

To conduct an in-depth inquiry, we designed a pragmatic constructivist case study using mixed methods (Merriam & Tisdell, 2015). This approach enables focus on a particular situation (Cleland et al, 2021), a large-scale virtual medical conference in this instance, to investigate participants’ perspectives using a variety of data sources. To develop good understanding of this case we interviewed conference organizers and surveyed delegates. The former effort informed survey development by gathering the intuitions of individuals with extensive conference experiences. The latter allowed more systemic data collection that generated quantitative data that could be submitted to cluster and factor analyses to query the existence of subgroups of participants and their defining characteristics.

### Context: case and setting

This study was conducted in conjunction with the first virtual European Respiratory Society (ERS) annual congress, which took place in September 2020 and had 29,020 international attendees. The congress itself is a once-a-year occasion for the world’s respiratory experts to gather, present, and discuss the latest scientific and clinical advances in respiratory medicine. Until 2019 the conference had occurred face-to-face.

### Key informant interviews

We conducted semi-structured interviews with stakeholders who had extensive conference experience to develop the survey items and better understand the variety of reasons for which delegates are perceived to attend conferences.

#### Participants and sampling

The ERS society consists of fourteen assemblies which are broad groups based on multidisciplinary specialist areas of interest within respiratory medicine (e.g., basic and translational science, respiratory clinical care and physiology, clinical imaging and paediatrics). We treated the heads of each assembly, secretaries and educational council chairs as the population of interest due to their extensive experience organising conferences and their multidisciplinary backgrounds. 23 individuals constitute this subgroup, which is drawn from a variety of countries (see Appendix 1). Invitations were sent to each individual by e-mail along with a brief description of the study and a consent form. Once an interview date and time was confirmed, the interview guide was sent to consenting participants.

#### Interview guide

Interviews focused on stakeholders’ professional background and experience, their perceptions of what motivates conference attendance, how motivations might change over time, and reactions to a list of motivations that were identified from the literature (see Appendix 2).

#### Data collection

Interviews were conducted using Zoom, audio‐recorded, and transcribed verbatim. Analytic memos were written during and after each interview and, after every third interview, the interviewer (SR) listened to the recordings, discussed preliminary thoughts with SH, and modified the interview guide to hone in on less developed issues.

#### Analysis of stakeholder interviews

Transcripts from the interviews were analysed using an iterative six-phase thematic analysis approach (Braun & Clarke, 2006): familiarisation with data, generating initial codes, searching for themes, reviewing themes, defining themes and writing them up. As codes and themes were identified, they were discussed by the authors and continually revised through iterative cycles of reflection, re-reading and re-writing, and re-negotiation.

#### Reflexivity

As constructivist researchers we aim to acknowledge both the emic (within setting) and the etic (outside setting) perspective of our research team. DSto, RC, NT, and CP are organizers for ERS and, hence, have personal viewpoints of what is most useful to congress attendees. SR is a PhD student who attended the first virtual ERS, but had no prior connection to the meeting. SH and KE are medical education researchers with extensive conference experience outside the ERS context. The group sought to manage their preconceptions by discussing each stage of the methodology and analyses while SR kept a reflective diary.

### Survey of attendees

#### Design

AMEE guide no. 87, developing questionnaires for educational research (Artino et al., 2014), was used to guide questionnaire design (see Appendix 3)*.* The demographic variables gathered included age, gender, country and workplace of practice and professional role. Other questions included number of previous ERS congress attendances, satisfaction with the virtual conference and future conference format preference. To address motivation, participants were asked to rate (using a 7-point Likert scale) 15 distinct reasons, drawn from the literature and key informant interviews, indicating why they usually attend the ERS congress and why they chose to attend the first virtual conference. The full survey is presented in Appendix 4, but free text responses (i.e., open-ended questions) were not included in our analysis.

#### Data collection

The survey was collated using SurveyMonkey (https://www.surveymonkey.com). Attendees were invited to participate, via email, after the conference. Two reminder emails were sent over the course of a month with an incentive to win a free registration to the ERS Congress 2021.

#### Analysis of survey

SPSS version 27 (SPSS, 2012) was used to analyse survey data. To explore the existence of subgroups, a k-means cluster analysis was performed. This process statistically groups respondents together in a manner that minimizes within-group variance and maximizes between-group variance according to selected variables. To define the variables that would be entered into the cluster analysis, questions about motivation were grouped using exploratory factor analysis. The suitability of the data was checked using the Kaiser–Meyer–Olkin measure for sample adequacy (KMO criterion), factors with Eigenvalue greater than 1 were extracted (Kaiser Criterion), and the resulting factors were rotated orthogonally according to the Varimax method. Cronbach’s alpha was then used to determine the internal consistency of the items contained in each factor and the scores from each item were averaged to create factor scores. The factor scores were then entered into the cluster analysis.

Although there were many clustering solutions with a variable number of groups generated, the final result presented in this paper was chosen based on the clustering that appeared to be most conceptually distinct (i.e., reflected clear differences between groups based on the ratings assigned to the motivational items presented). This was done in line with the aim of this study, to help conference organizers optimize the targeting of sessions towards each cluster. For example, if a group primarily scored high in one single factor associated with learning motivation, that could guide thinking about how to focus sessions that best address those attendees’ learning needs.

To examine the relationship between cluster assignments and demographics (research question 2), we performed Chi-squared tests comparing the proportion of participants that fell into each cluster as a function of the following variables: gender, age, conference experience, satisfaction, and future conference format preference.

## Results

### Key informant interviews

Of the 23 stakeholders who were invited for interview, 13 responded and consented to participate. Their interviews lasted approximately 33 min on average with 7 countries and 9 different professional roles/specialties represented (Appendix 1). From the thematic analysis conducted, we identified two main themes regarding what participants thought likely to influence the motivations of attendees: (1) length of time/depth of specialising in a field and (2) engagement with past conferences (see Table 1). In general, the results from the thematic analysis demonstrated that participants expected motivations to change from being broad-based early in one’s career/specialisation to having a more prioritised focus. For example, younger or newly specialised attendees were expected to value topic overview sessions whereas those who were in the field longer were expected to want very high-level content describing the latest scientific findings in a field. Similarly, participants indicated that initial engagement with conferences is most likely to occur for external rewards (e.g., CPD points, meeting new people and disseminating work), whereas the more conferences one has attended the more motivations were expected to transition away from learning to interacting with others (professional colleagues and friends). As this transition occurs, participants expected delegates to draw increasing amounts of motivation from gains in personal enjoyment (e.g., some time away from clinical realities).

**Table 1:** Themes from thematic analysis of key informant interviews

Themes	Corresponding quotes
Theme 1: with further specialisation in a field, motivations for conference attendance change from being broad to more focussed	*“just started in paediatric pulmonology, you will go to the big sessions, getting an overview of the field, *etc*. And if you have more experience,[…] you try to get new information by going to sessions that you are not used to going to.*”—**Stakeholder 13** “*If you are very highly specialised, when you go to a conference, you just go for a very high content, you know what you want […]. If you are not specialised you maybe prefer more skill sessions or educational sessions.”*—**Stakeholder 6** “*It’s more broader as a student; you’re more interested in getting, drawing attention to your work, getting to know new people. It’s a different thing.*”—**Stakeholder 5**
Theme 2: as engagement with conferences increases, there is a shift away from high level content learning to social motivations	“*The motivation to learn of course tends to decrease while what increases is the motivation to go to the congress in order to consolidate already established networks*”—**Stakeholder 7** “*I don’t need the CPD points. Previously I might have wanted to collect a few, but now I certainly don’t need any. So, probably I go for the academic and I go to meet my friends.*”—**Stakeholder 1** “*an academic conference give us, or gives me at least, some space from the clinical reality, from patients and from calls and from people*.”—**Stakeholder 4**

### Survey of attendees

#### Demographics

1,229 individuals completed the survey for a response rate of 4.2%. 51% of respondents were from Europe (n = 630), 18% Asia (n = 217), 9% South America (n = 111), 5% North America (n = 58), 3% Oceania (n = 38) and 3% Africa (n = 34); 141 (11%) did not specify a region. 52% (n = 639) reported being male and participants’ age was normally distributed with a peak in the 41–45-year-old range (see Appendix 5). The most common workplace (n = 477; 38.8%) was a university hospital (see Appendix 6).

*Research question 1:* Do attendees’ motivations for conference attendance cluster together into subgroups of participants?

The factor structure for questions about why respondents usually attend in-person ERS conferences and why they chose to attend the first virtual meeting were highly similar, so only the latter analysis is presented, in Table 2. The KMO measure of sample adequacy was 0.83, signalling good suitability of the data for factor analysis. Upon varimax rotation, 3 factors were found to explain 59.1% of the total variance. The factors were named ‘**personal goals’** (7 questions), ‘**social goals’** (4 questions) and ‘**learning goals**’ (2 questions). Two questions could not be clearly assigned to a factor: “*to support the career development of others*” and “*to support my career development*”. Table 2 illustrates factor loadings and internal consistencies for each factor.

**Table 2:** Rotated component matrix factor analysis results for respondents’ ratings of their motivations for attendance of first virtual ERS congress

Motivation for virtual attendance	Social	Personal	Learning
*To make/deepen professional connections*	**0.837**		
*To socially interact and spend time with peers*	**0.801**	0.355	
*To present my scientific/academic work*	**0.737**		
*To meet experts and leaders in the field*	**0.696**		0.369
*To improve my practical clinical skills (e.g. online live bronchoscopy procedure)*	**0.547**	0.499	
*To support career developments of others*	**0.531**	0.452	
*To foster personal change*		**0.861**	
*To foster change in my workplace*		**0.854**	
*To fulfil the requirements of professional certification bodies, such as attaining CPD/CME credits*		**0.629**	
*To improve my teaching skills*	0.489	**0.615**	
*To improve my communication skills (e.g., patient communication, team communication)*	0.586	0.595	
*To support my career development*		0.416	0.395
*To learn the latest scientific findings*			**0.902**
*To learn the latest advancements in patient care*			**0.848**
Internal consistency (Cronbach’s alpha)	0.89	0.82	0.82

**Fig. 1:**
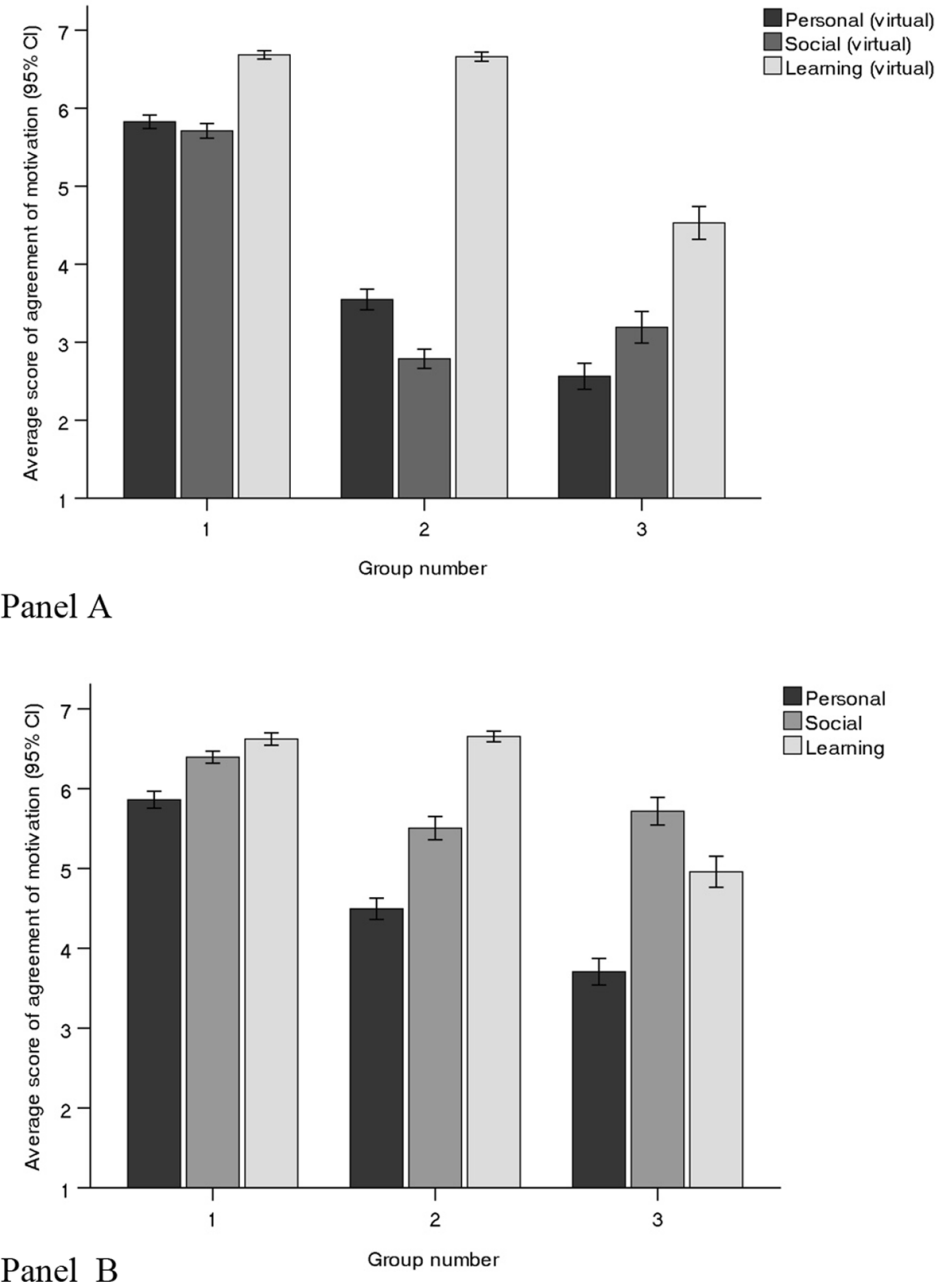
Average factor scores for subgroups based on cluster analysis (Panel A = motivations for “this virtual conference”; Panel B = motivations for “the usual in person conference”)

Using factor scores for either “in-person” or “this virtual conference” scores, cluster analyses revealed three subgroups of participants (see Fig. 1). The largest group (n = 500), Group 1, scored high on all three factors. Group 2 (n = 349) scored high on ‘learning’ but were less motivated by social and personal factors. Group 3 (n = 188) showed less consistency between formats, scoring highest for the social factor when asked about in-person conferences and highest on the learning factor when asked about virtual meetings. 192 participants could not be classified due to missing values in one of the factor variables.

*Research question 2:* Are subgroups associated with attendee demographics, experiences with past in-person conference attendance, satisfaction with virtual conference formats, or preference for future conferences?

Table 3 illustrates that subgroup membership was not related to age or gender, but was associated with differences in prior conference attendance, satisfaction with the virtual meeting, and future conference format preferences. In terms of prior conference attendance, Table 3 illustrates that Group 1 was least experienced and Group 3 was most experienced. In turn, Group 1 was most satisfied with the virtual conference format and Group 3 was least satisfied. Despite that difference, all three groups most preferred a hybrid format (combined in-person and virtual) for future meetings. If a hybrid format is ruled out, Group 3 was most inclined towards a return to in-person conferences.

**Table 3:** Statistical relationships between variables and group membership

Variable	Group 1	Group 2	Group 3	Overall	Statistics
*Gender*					
Female	253 (56.9%)	171 (53.3%)	83 (47.7%)	507 (53.9%)	${\chi }_{df=2}^{2}=4.3, p=0.1$
Male	192 (43.1%)	150 (46.7%)	91 (52.3%)	433 (46.1%)	
*Age*					
< 36	99 (22.1%)	57 (17.6%)	41 (23.3%)	197 (20.8%)	${\chi }_{df=6}^{2}=6.9, p=0.3$
36–45	149 (33.3%)	98 (30.3%)	47 (26.7%)	294 (31.1%	
46–55	103 (23.0%)	86 (26.6%)	43 (24.4%)	232 (24.5%)	
> 55	96 (21.5%)	82 (25.4%)	45 (25.6%)	223 (23.6%)	
*# of previous attendances*					
0	206 (41.2%)	75 (21.5%)	28 (14.9%)	309 (29.8%)	${\chi }_{df=4}^{2}=75.4, p<0.001$
1–9	232 (46.4%)	227 (65.0%)	111 (59.0%)	570 (55.0%)	
10 or more	62 (12.4%)	47 (13.5%)	49 (26.1%)	158 (15.2%)	
*Satisfaction with virtual conference*					
1–3	28 (5.6%)	29 (8.3%)	33 (17.6%)	90 (8.7%)	${\chi }_{df=4}^{2}=72.8, p<0.001$
4–5	129 (25.8%)	141 (40.4%)	87 (46.3%)	357 (34.4%)	
6–7	343 (68.6%)	179 (51.3%)	68 (36.2%)	590 (56.9%)	
*Preference for future conferences*					
Online	84 (16.8%)	77 (22.1%)	14 (7.4%)	175 (16.9%)	${\chi }_{df=6}^{2}=29.727, p<0.001$
In-person	95 (19.0%)	67 (19.2%)	61 (32.4%)	223 (21.5%)	
Combination	313 (62.6%)	199 (57.0%)	111 (59.0%)	623 (60.1%)	
No preference	8 (1.6%)	6 (1.7%)	2 (1.1%)	16 (1.5%)	

## Discussion

Our study identified three groups of medical conference attendees that could be clustered based on their motivations for attendance (i.e., learning factor, personal factor and social factor). Group 1 was motivated by all factors whereas Group 2 was mainly motivated by the learning factor. Group 3 reported drawing greatest motivation from the social factor when considering in-person conferences and the learning factor when considering virtual meetings. Before discussing the implications of these observations, we note that while demographics of age and gender were unrelated to group membership, Group 1 tended to be least experienced and most satisfied with the virtual format whereas the opposite was true for Group 3. All three groups expressed preference for hybrid conferences in the future; if hybrid conferences are not an option, Group 3 was notably more inclined towards in-person conferences. The apparent relationship between group membership and experience is consistent with our stakeholder interviews, which suggested that attendees’ motivations would be conditional upon their engagement with conferences. Knowing about these differences can help make conference attendance more appealing by better enabling organizers to meet participants’ needs. They might also help potential delegates better reflect on their priorities and how well a conference is likely to fulfil their desires.

In that regard, the first thing of note is that nearly 2/3rds of respondents in all groups expressed a preference for meetings that combined virtual and in-person activities. This observation will be particularly challenging for conference organizers because it has become well known that hybrid meetings raise costs. Anecdotally, the organizers of two major conferences have reported, separately and independently, to one of our authors that they effectively had to pay to organize two meetings at once. Beyond the obvious impact on sustainability, determining whether or not the additional cost is money well spent will be dependent on the degree to which conferences are built in ways that fulfil delegates’ motivations for attending. Doing so will be achievable only if organizers understand the degree to which subgroups of attendees with differing desires are present.

In this study, Group 1, comprised 40.7% of the sample, attended conferences for learning, personal and social motivations, and tended to have attended fewer conferences than other delegates. The latter observation means these attendees have less basis for comparison, which might have contributed to their higher satisfaction with the virtual conference. The broad range of motivations amongst those with fewer conference attendances was also highlighted by our stakeholders’ interviews. For conference organizers, the multidirectional focus of this large group suggests it to be useful to focus on designing an array of sessions. For example, this group expressed largely equal desires to learn about the latest advancements in their field, deepen their social connections and achieve their personal goals. These motivations are consistent with previous reports that conferences can help build the reputations of scientists (Moynihan, 2008), inducing many organizers to include sessions on how to network and collaborate effectively (e.g., how to join a conference committee (Hartsell-Gundy, 2019) or acquire a mentor) as well as sessions on how to present one’s own academic work. How to best balance such activity with traditional information delivery sessions across conference formats, however, remains to be determined. We think it likely that organizers would benefit from planning their hybrid conference to more deliberately focus in-person networking sessions on those who are newer to the conference, enabling them to join the community and improve their engagement, while holding virtual sessions for moments aimed more predominantly at knowledge building, which may not be so dependent on the benefits of face-to-face contact. Similarly, simulation laboratories that are known to offer learning on how to recognise and treat complex clinical problems (Good, 2003) may be best divided across conference formats in a manner that pairs online information sharing with use of limited “in-person” time for practical clinical skills sessions aimed at more directly transmitting expertise or fine-tuning these applied skills. The combination of activity across hybrid formats, in other words, might more thoughtfully be determined by when and how interacting with others in-person is most likely to foster personal change that can be applied to the workplace rather than assuming that all sessions can proceed equally effectively in-person or through virtual mediums.

Group 2, comprising 28.1% of the sample, reported attending conferences primarily for learning motivations with much less emphasis on personal or social motivations. In other words, they were predominantly interested in learning “the latest scientific findings” and “the latest advancements in patient care” (Table 2). Stakeholder interviews suggested such a pattern largely to be anticipated for individuals who are further specialising and, thereby, require a high level scientific content. Group membership was not associated with age (which would be expected to be associated with degree of pursuing further specialization), in our study, thereby, reinforcing the importance of empirically exploring attendee motivations, rather than relying on stakeholder intuitions. This group was more likely than Group 3, the most experienced group, to rate virtual formats as preferable, prompting further reflection on what evidence exists regarding the benefits and limitations of virtual learning. Studies outside of medicine conclude that virtual conferences provide better file sharing, data presentation, and virtual interfaces for speakers and attendees (Sarabipour, 2020). In addition, those with socioeconomic limitations, geographic limitations, and researchers with family commitments and responsibilities may prefer attending virtual events (Sarabipour, 2020). In line with accommodating this group, conference organizers should note that virtual conferences are likely to be valued for their accessibility (i.e., having learning resources available on-demand, online and for a longer time period) as they may provide opportunity for a much broader scope of sharing and, thus, learning from conferences (Lortie, 2020). We urge caution because potential delegates generally seem less interested in paying for virtual meetings despite organizers knowing them to have considerable cost. Including virtual components that are available for longer periods, however, may offset greater expense by attracting more individuals compared to conferences that require in-person attendance and are, hence, time limited. Relying on such activity again requires careful contemplation about what sessions are likely to be “evergreen” in the sense of being equally meaningful when engaged asynchronously.

Finally, the third cluster of delegates, comprising 15.3% of the sample, was particularly intriguing because its motivations were most discrepant across virtual vs in-person formats. They rated the learning factor highest in the former and the social factor highest in the latter. Of the minority who did not prefer virtual formats, this group was relatively inclined towards in-person conferences for the future, but discrepancy noted suggests that they would approach in-person and virtual meetings with different motivations. While smaller in overall number, this group was most experienced with conferencing, suggesting that conference organizers should be careful about making decisions based on head count alone; a smaller group that attends regularly might have a higher impact on the conference and long-term revenue than a larger group that is not as focused on conferencing. Stakeholder interviews suggested that needs became more narrowed and centre on socialising as the number of conference attendances increase, but our survey data suggest that likely stakeholder impressions have historically been built predominantly on experience with in-person meetings. While previous work has suggested that the overall value rating assigned to virtual conferences is independent of participants’ perceived importance of social interaction (Seidenberg et al, 2021), the lower satisfaction ratings Group 3 gave to the new virtual ERS conference raises question of whether other factors such as previously having attended many in-person conferences changes the way the value of interaction is perceived. That is, if an individual has attended many times, perhaps they have lower expectations regarding social factor activities within virtual meetings. For organizers, knowing that their most dedicated delegates are more likely to value in-person activity could suggest a need to prioritize in-person networking and mentoring sessions for more than just the benefit they might bring to newer members looking to join their community. It also suggests value to organizing more informal opportunities (Roos et al, 2020), in an effort to maintain the group’s attention while creating social connections that delegates with less experience may have not had an opportunity to learn to appreciate.

It is noteworthy that our study did not find age or gender differences across subgroup. Although there is no literature within medicine, others have reported that personal safety motivations are more important to women than men and that younger attendees rate professional development opportunities higher (Mair, 2010). Our study did not focus on logistical aspects of conferencing including consideration of physical location and disruption to things like personal lives that might be particularly impactful for those with young families. Given these differences and the fact that previous work focused upon in-person meetings (Mair, 2010), considerations of gender and age may require further investigation if hybrid conferences again become more prominent.

### Strengths and limitations

Strengths of this study include exploration of these issues at an international conference that was conducted virtually for the first time, thereby enabling a large and diverse sample while also taking advantage of a novel opportunity for comparison to the literature that has built up around previous in-person conferences. Limitations include that the response rate of the survey was low (4.2%). The overall number of responses was high but the results generated might also be limited by response biases attributed to this being the first virtual congress and selection bias (as participants became eligible only by virtue of having chosen to attend the virtual conference). That is, we are missing the perspectives of those who did not attend the conference but normally would have, thus raising the possibility that even more subgroups than those identified here might exist.

Future research should be conducted to replicate this study to investigate if the same clusters can be elicited in other contexts. In addition, asking respondents more details about their stage of specialisation might yield further insights, from the attendees’ perspective, of any relationships between clusters of delegates and their motivations for attendance.

## Conclusion

Our survey identified three distinct groups of conference attendees who were differentiable based on their motivations for conference attendance. In both in-person and virtual conferences, *Group 1* was motivated by learning, social and personal factors whereas *Group 2* was motivated mainly by the learning factor. *Group 3*’s motivation was more variable, focusing on the social factor for in-person conferences and the learning factor for the virtual conferences. Group number was associated with increasing amounts of conference experience, as also suggested by stakeholder interviews; and declining level of satisfaction with the virtual conferences. 2/3rds of all groups preferred that future conferences be hybrid in nature. The identification of these groupings can be used to optimize the alignment between conference format and goal fulfilment by offering guidance regarding what needs should be prioritized and how one might arrange meetings to satisfy motivations.

## Ethical Approval

The study was deemed exempt from ethical review after application to the Regional Ethics Committee of the Canton of Bern (member of the Swiss Association of Research Ethics Committees, Switzerland) BASEC-Nr: Req-2020–00,771 (Acquired: 19/06/2020). Confidentiality and anonymity of speaker interview data and all evaluation survey data was maintained throughout the study, including removal of identifying information from quotations.

## Appendices

### Appendix 1

Demographic stakeholder interview participant data with multidisciplinary professional role and country distribution.

**Table:**

1	GP (UK)
2	Pulmonologist and Intensivist (Netherlands)
3	Sustaining Professor of Respiratory Medicine (Greece)
4	Adult Respiratory Physician (Switzerland)
5	Respiratory Physiotherapist (Netherlands)
6	Adult Respiratory Physician (Italy)
7	Professor in Biomedical Engineering (Italy)
8	Adult Respiratory Physician (Germany)
9	Adult Respiratory Physician (UK)
10	Professor in Pharmacology (Netherlands)
11	Scientist (Switzerland)
12	Paediatric Respiratory Physician (Austria)
13	Paediatric Respiratory Physician (Netherlands)

### Appendix 2

Interview guide for ERS stakeholders

Thank you for kindly agreeing to take part in this interview.

The main objective of this interview is to gain a better understanding of ERS stakeholders’ role within the ERS, their personal experiences of attending conferences, their judgement of why they believe participants attend the ERS conference and also their ideas on the impact of turning the conference to an online format. The interview will be recorded and transcribed verbatim for analysis. All data will remain anonymous.

Thank you for your time and thoughts.

Intro and personal experiences

1. What is your professional background?
2. Please tell me about your role in the ERS?
3. What has your role been, specifically related to organising past ERS conferences?
4. What motivated you to become actively involved in organising ERS conferences?

Attendees needs

1. When you think about academic conferences, what do you consider to be the main motivations for attending?
  - Probe: That is, what do people look for in a conference/why do they attend?
2. What factors influence which of these motivations takes priority?
  - Probe: For example, have you found that the length of time in a field changes the things you look for in a conference?
3. Is there anything different about the ERS annual congress relative to what you have just said about conference attendance in general?
  - Probe: What do you think are the main reasons for visiting the ERS annual congress?
  - Probe: What factors do you think influence the reasons for attending the ERS annual congress? e.g. different professional backgrounds, different career stages etc.

Survey regarding reasons why participants come to the ERS annual congress

We envision to giving participants the following options to choose from, on why they attend the ERS congress.

1. What are your thoughts on these? Are there any missing?
  - Probe: Do you think they are likely motivations? Do you think they are likely to motivate only a few people? Does the list prompt you to think of other things that may be overlooked?
    1. **I attend the congress to learn the latest biomedical scientific discoveries**
    2. **I attend the congress to learn the latest advancements for clinical practice (e.g. guidelines for evidence-based practice)**
    3. **I attend the congress to learn and practice clinical skills (e.g. practical skills, communication skills, technological skills like bronchoscopy)**
    4. **I attend the congress to make or deepen networking connections for collaboration**
    5. **I attend the congress to support presenting and publishing my scientific and academic work**
    6. **I attend the congress to improve my teaching/ supervising skills**
    7. **I attend the congress to support my career**
    8. **I attend the congress to meet experts/role-models to help support my career**
    9. **I attend the congress to be better able to foster change in my organisation (e.g. implementation of latest safety preventative measures)**
    10. **I attend the congress to improve my well-being (e.g. work-life balance)**
    11. **I attend the congress to interact and spend time with peers and colleagues**
    12. **I attend the congress as an opportunity to explore a new city**
2. Do you think these reasons are considered when planning the ERS annual congress? If so, how?
  - Probe: Has there historically been anything that you felt to be missing from the ERS annual congress (i.e., desires attendees have that the conference doesn’t fulfil? If so, what has prevented the ERS from addressing these needs?)

### Appendix 3

Stages followed during survey design

**Table:**

Design stages and purpose	Description of how we conducted each step
1. Literature review to ensure alignment with relevant prior research on conference motivations	The study uses motivational items relevant from pre-existing literature review within the tourism and business literature (Mair, 2010; Mair & Frew, 2018). Relevant to the medical context, a scoping review of the 14 broad impact categories for CPD identified by Allen et al. (2019), was used to further develop this with proxy items to represent each factor. Additional items were also included, such as the item “to explore a new location,” derived from Mair’s (2010). The initial list of motivational items at this stage was twelve.
2. Interviews to learn how the population conceptualises and describes the list of motivations	Author SR conducted semi-structured interviews with thirteen ERS stakeholders who had extensive conference attendance experience were asked what they believed were the motivations for attendees’ conference attendance and their thoughts on the list of 12 possible motivations that were elicited in Step 1. For further details on the sampling, conduction and analysis of the interview data, please see the written interview methods section.
3. Findings synthesised from literature and interviews and sent to experts in the field	“*To fulfil the requirements of professional certification bodies, such as attaining CPD/CME credits*” was deemed an important addition after the stakeholder interviews. Separating "*supporting my own career*” and “*supporting the career of others*” was also noted. In addition, communication skills being included as an additional item as opposed to within clinical skills was highlighted. A revised questionnaire was sent to educational experts for review.
4. Develop questions and ensure they are clear, understandable and written in accordance with current best practices in survey design	Culmination of literature, stakeholder interview data and expert opinion resulted in a list of 15 items. “To explore the city/region where the conference is held” was removed for virtual context questions. Although using a Likert-type scale is suggested to be avoided, we choose to ask respondents to rate the importance of the motivational items on a Likert-type scale from 1 (strongly disagree) to 7 (strongly agree) for retrospective accounts on previous in-person congresses and the virtual congress which they had attended at the time of the study. We believed this to be suitable, as during the stakeholder interviews, 4 out of the 13 responded spontaneously to the list by rating their agreement to the statement out of 10.
5. Conduct expert validation to assess how clear and relevant the questions are	“To foster change in my organisation (e.g., implementation of latest safety preventative measures)” was revised to “To foster organisational change” after additional feedback from an interdisciplinary group of educational experts and an English language expert.
6. Conduct cognitive interviews to ensure that respondents interpret questions in the manner that survey designer intends	As a final step, three cognitive interviews were conducted with conference attendees who were multidisciplinary and at different stages of their career. This was to check whether all the items were understandable and reassess how long the survey would take. E.g., “to foster organisational change” was changed to “to foster change in my workplace”.

### Appendix 4

Survey distributed to conference delegates

**Part 1: virtual ERS international congress 2020**

*Thinking about the difference between* ***previous in-person*** *ERS International Congresses with the* ***virtual ERS International Congress 2020:***

1. How satisfied were you by the **virtual** ERS International Congress 2020 overall? Not at all satisfied (1) to Completely satisfied (7).
2. How many times have you attended the ERS International Congress **in person**?
  - Never
  - 1
  - 2–4
  - 5–9
  - 10–19
  - 20 or more
3. What worked well in the virtual conference compared to in-person conferences? (Free Text)
4. What, if anything, prevented achievement of your needs with this virtual conference, compared with past in-person conferences? (Free text)
5. How do you think the virtual conference could be improved? (Free Text)
6. Which format would you prefer in the future?
  - Online
  - In person
  - A combination of both
  - No preference
7. What is the main reason for your preferred conference format? (Free text)

**Thank you very much for your answers! The second part of this questionnaire will ask more specific questions about your conference needs, expectations and reasons of attendance. It will take approximately 8 min to complete it. Your answers will help us shape and develop the next ERS Congress 2021. By completing this survey entirely, you could win one of five free online registrations offered for next year’s ERS Congress.**

**YES I would like to contribute**

**NO (SKIP to part 7—demographic data)**

**Part 2: past experiences**

8. **Prior to 2020**, how many times have you taken part in the ERS International Congress **online**?
  - Never
  - 1
  - 2
  - 3
  - 4

**Part 3: reasons for attending an ERS international congress in person**

9. Why do you **usually** attend the ERS International Congress **in person**? *Rate the statements below from strongly disagree (1) to strongly agree (7) or Not Applicable (N/A).*

I **usually** attend the ERS International Congress:

- **to support ****my**** career development**
- **to support career developments of ****others**
- **to socially interact and spend time with peers**
- **to present my scientific/academic work**
- **to meet experts and leaders in the field**
- **to make/deepen professional connections**
- **to learn the latest scientific findings**
- **to learn the latest advancements in patient care**
- **to improve my practical clinical skills** (e.g. in-person bronchoscopy)
- **to improve my communication skills**
- **to improve my teaching skills**
- **to fulfil the requirements of professional certification bodies, such as attaining CPD/CME credits**
- **to foster personal change**
- **to foster change in my workplace**
- **to explore the city/region where the conference is held**

**Part 4: reasons for attending this year ERS international congress**

10. Why did you take part in the **virtual ERS International Congress 2020**? *Rate the statements below from strongly disagree (1) to strongly agree (7 or Not Applicable (N/A).*

I attended **this year’s virtual conference**:

- **to support ****my**** career development**
- **to support career developments of ****others**
- **to socially interact and spend time with peers**
- **to present my scientific/academic work**
- **to meet experts and leaders in the field**
- **to make/deepen professional connections**
- **to learn the latest scientific findings**
- **to learn the latest advancements in patient care**
- **to improve my practical clinical skills (e.g. online live bronchoscopy procedure)**
- **to improve my communication skills**
- **to improve my teaching skills**
- **to fulfil the requirements of professional certification bodies, such as attaining CPD/CME credits**
- **to foster personal change**
- **to foster change in my workplace**

11 How well were the following reasons fulfilled by this year’s virtual conference?
  (closed question:—Not at all fulfilled (1)–completely fulfilled (7), plus “Not applicable” (N/A)).
  - **to support ****my**** career development**
  - **to support career developments of ****others**
  - **to socially interact and spend time with peers**
  - **to present my scientific/academic work**
  - **to meet experts and leaders in the field**
  - **to make/deepen professional connections**
  - **to learn the latest scientific findings**
  - **to learn the latest advancements in patient care**
  - **to improve my practical clinical skills (e.g. online live bronchoscopy procedure)**
  - **to improve my communication skills**
  - **to improve my teaching skills**
  - **to fulfil the requirements of professional certification bodies, such as attaining CPD/CME credits**
  - **to foster personal change**
  - **to foster change in my workplace**

**Part 5: reasons why you attend an ERS international congress**

**In order for us to understand how we can shape future conferences in line with your interests, we want to learn more about two key reasons for participating in the virtual ERS International Congress 2020.**

12. Please choose **one** reason for taking part in the virtual conference, which you would like to share your thoughts on:
  - **to support my career development**
  - **to support career developments of others**
  - **to socially interact and spend time with peers**
  - **to present my scientific/academic work**
  - **to meet experts and leaders in the field**
  - **to make/deepen professional connections**
  - **to learn the latest scientific findings**
  - **to learn the latest advancements in patient care**
  - **to improve my practical clinical skills (e.g. online live bronchoscopy procedure)**
  - **to improve my communication skills**
  - **to improve my teaching skills**
  - **to fulfil the requirements of professional certification bodies, such as attaining CPD/CME credits**
  - **to foster personal change**
  - **to foster change in my workplace**
13. What aspects of the virtual conference helped fulfil your chosen reason (for attendance)? (Free text)
14. How could the virtual conference be improved for your chosen reason? (Free text)
15. Please choose **a second reason** for attending on which you would like to comment on:
  - **to support ****my**** career development**
  - **to support career developments of ****others**
  - **to socially interact and spend time with peers**
  - **to present my scientific/academic work**
  - **to meet experts and leaders in the field**
  - **to make/deepen professional connections**
  - **to learn the latest scientific findings**
  - **to learn the latest advancements in patient care**
  - **to improve my practical clinical skills (e.g. online live bronchoscopy procedure)**
  - **to improve my teaching skills**
  - **to improve my communication skills**
  - **to fulfil the requirements of professional certification bodies, such as attaining CPD/CME credits**
  - **to foster personal change**
  - **to foster change in my workplace**
  - **Other (please specify)**
  - **No other reason (Skip to part 6)**
16. What aspects of the virtual conference helped fulfil your chosen reason? (Free text)
17. How could the virtual conference be improved for your chosen reason? (Free text)
18. Would you like to comment on any other reason?

- Yes- (continue to Q19)
- No- (skip to part 6).

19. Please choose a third reason for attending on which you would like to comment further:
  - **to support ****my**** career development**
  - **to support career developments of ****others**
  - **to socially interact and spend time with peers**
  - **to present my scientific/academic work**
  - **to meet experts and leaders in the field**
  - **to make/deepen professional connections**
  - **to learn the latest scientific findings**
  - **to learn the latest advancements in patient care**
  - **to improve my practical clinical skills (e.g. online live bronchoscopy procedure)**
  - **to improve my communication skills**
  - **to improve my teaching skills**
  - **to fulfil the requirements of professional certification bodies, such as attaining CPD/CME credits**
  - **to foster personal change**
  - **to foster change in my workplace**
  - **Other (please specify)**
20. What aspects of the virtual conference helped fulfil your chosen reason? (Free text)
21. How could the virtual conference be improved for your chosen reason? (Free text)

**Part 6: areas for conference improvement**

22. Do you have any further suggestions on how the annual ERS International Congress can better address your needs?

(Free text)…

**Part 7: basic demographic data**

For the final section, we would like to learn more about you and your professional role. Please kindly answer the few questions below.

**Age:**

- < 20
  21–25
- 26–30
  31–35
- 36–40
  41–45
- 46–50
  51–55
- 56–60
   61–65
- 66–70  
  > 70

**Gender:**

- Male
- Female
- Prefer not to say

**Country of practice:**

*Drop-down menu*

**Professional role:**

(Please select all that apply and give further details in “Other” section)

- Adult Pulmonologist/Clinician
- Clinical Researcher
- General Practitioner
- Journalist
- Medical Student
- Medical Technical Assistant
- Nurses
- Paediatrician
- Pathologist
- Patient
- Physician in Pulmonary Training
- Physiologist
- Radiologist
- Respiratory Critical Care Physician
- Respiratory Physiotherapists
- Respiratory Therapists
- Sales, Marketing, Industry
- Scientist (basic, translational)
- Thoracic Oncologist
- Thoracic Surgeon
- Other

**Please write your job title (e.g. consultant in respiratory medicine) (free text box)**

**Number of years of Practice (post-graduation)** *(scale with 1- 70).*

**Place of Practice**

- Academic institution
- University hospital
- Non-university hospital
- Private/Independent
- Industry
- Governmental organisation
- Non-governmental organisation
- Other… (free text)

### Appendix 5

Distribution of survey respondents’ age ranges

**Table:**

Age ranges (Years)	No. of attendees
21–25	11
26–30	65
31–35	138
36–40	156
41–45	174
46–50	152
51–55	128
56–60	133
61–65	79
66–70	42
> 70	15

### Appendix 6

Distribution of survey respondents’ place of work

**Table:**

Place of work	No. of participants
Non-government Organisation	23
Other	40
Industry	72
Government organisation	99
Private/independent	198
Non-university hospital	228
Academic institution	288
University hospital	477
